# A new species of *Eusirus* from Jeju Island, Korea (Crustacea, Amphipoda, Eusiridae)

**DOI:** 10.3897/zookeys.640.10630

**Published:** 2016-12-13

**Authors:** Tae Won Jung, Min-Seop Kim, Ho-Young Soh, Seong Myeong Yoon

**Affiliations:** 1 National Marine Biodiversity Institute of Korea, Seocheon 33662, Korea; 2 Faculty of Marine Technology, Chonnam National University, Yeosu 59626, Korea; 3 Department of Biology, Chosun University, Gwangju 61452, Korea

**Keywords:** Amphipoda, eusirids, Eusirus bulbodigitus, Korea, new species, taxonomy

## Abstract

A new eusirid amphipod, *Eusirus bulbodigitus* **sp. n.**, from Jeju Island, Korea is described with a detailed description and illustrations. *Eusirus bulbodigitus* **sp. n.** shows common features with the five known eusirid amphipods *Eusirus abyssi* Stephensen, 1944, *Eusirus columbianus* Bousfield & Hendrycks, 1995, *Eusirus hirayamae* Bousfield & Hendrycks, 1995, *Eusirus laticarpus* Chevreux, 1906, and *Eusirus parvus* Pirlot, 1934, such as the mandibular palp article 3 bearing a group of setae laterally. However, this new species is differentiated by the combination of the following characteristics: the eyes are poorly developed, the propodus on pereopod 4 is slightly shorter, the inner margin of dactylus on pereopod 4 is swollen, the length of pereopods 5–7 is moderate, the urosomite 1 has a dorsal protrusion distally, and the telson is shallowly cleft. This is the first record of the genus *Eusirus* Krøyer, 1845 from Korean waters.

## Introduction

Members of the genus *Eusirus* Krøyer, 1845 share several synapomorphies such as the raptorial gnathopods having a lobate carpus and enlarged propodus, well-developed molars, strong and dentate incisors and dentate left lacinia on the mandible, and a strong maxillipedal palp (Bousfield 1978, Barnard and Karaman 1991, Bousfield and Hendrycks 1995). Up to date, this genus contains 27 nominate species worldwide, but the further taxonomic study on the validity of them is needed (Sars 1895, Chevreux 1906, 1911, 1912, Stephensen 1912, 1944, Pirlot 1934, Gurjanova 1951, Schellenberg 1955, Birstein and Vinogradov 1960, Barnard 1961, Andres 1979, Bousfield and Hendrycks 1995, Andres et al. 2002). In the North Pacific, Bousfield and Hendrycks (1995) reviewed the systematics and distributions of the family Eusiridae Stebbing, 1888, but our taxonomic knowledge concerning the fauna of the genus *Eusirus* still remains very poor with the records of six species only: *Eusirus cuspidatus* Krøyer, 1845 from Alaska and the Bering Sea (Gurjanova 1951, Shoemaker 1955, Bousfield and Hendrycks 1995); *Eusirus columbianus* Bousfield & Hendrycks, 1995 from Alaska and British Columbia (Bousfield and Hendrycks 1995); *Eusirus hirayamae* Bousfield & Hendrycks, 1995 from Japan (Hirayama 1985, Bousfield and Hendrycks 1995); and *Eusirus bathybius* Schellenberg, 1955, *Eusirus fagilis* Birstein & Vinogradov, 1960 and *Eusirus parvus* Pirlot, 1934 from western Pacific regions close to the Equator (Pirlot 1934, Schellenberg 1955, Birstein and Vinogradov 1960, Bousfield and Hendrycks 1995).

Here, we report a new eusirid species, *Eusirus bulbodigitus* sp. n. from Jeju Island, Korea, belonging to the family Eusiridae with a detailed description and illustrations. This is the first record of the genus *Eusirus* from Korean waters.

## Material and methods

Sample was collected from subtidal zone using a sledge net (mesh size 300 µm, mouth size 120 × 45 cm). Specimen was initially fixed with 5% formaldehyde-seawater solution and then preserved with 85% ethyl alcohol after sorting in the laboratory. It was stained with lignin pink dyes. Appendages were dissected in Petri dishes filled with glycerol using dissection forceps and a needle under a stereomicroscope (SZH10; Olympus, Tokyo, Japan). Its appendages were mounted on permanent slides using polyvinyl lactophenol solution. Drawings were made under a light microscope (LABOPHOT-2; Nikon, Tokyo) with the aid of a drawing tube. Definition of the term for ‘seta’ and its types follows those of Watling (1989). Type material was deposited at the National Institute of Biological Resources (NIBR), Incheon, Korea.

## Systematic account

### Order Amphipoda Latreille, 1816 Suborder Gammaridea Latreille, 1802 Family Eusiridae Stebbing, 1888 Genus *Eusirus* Krøyer, 1845

#### Eusirus bulbodigitus sp. n.

Taxon classificationAnimaliaAmphipodaEusiridae

http://zoobank.org/F4F288E6-5704-4F02-BD55-B3ADC5AABE13

**Figure 1.**
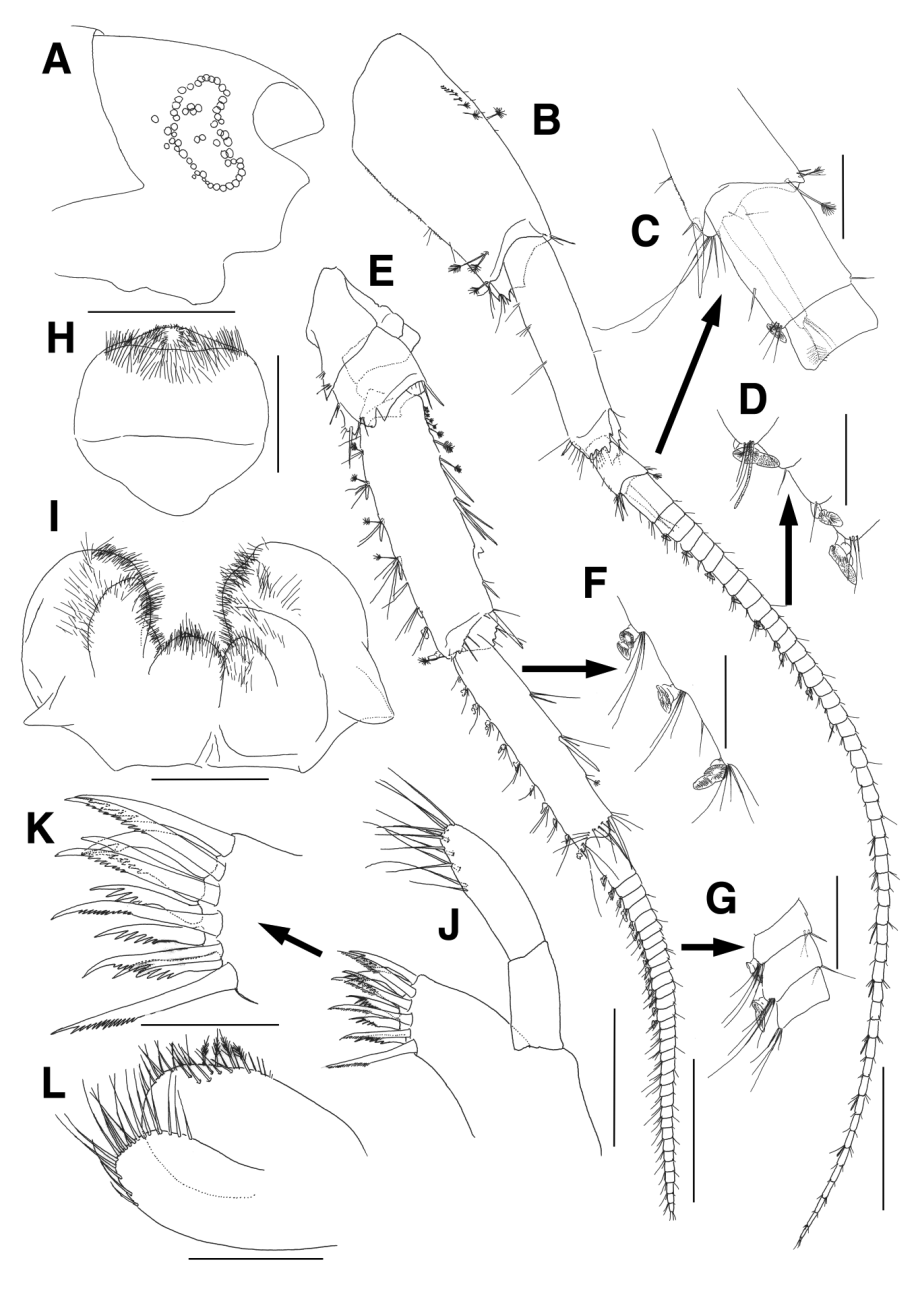
*Eusirus bulbodigitus* sp. n., holotype, male, NIBRIV0000332003, 11.3 mm. Jeju Island, South Korea. **A** Head **B** Antenna 1 **C** Accessory flagellum **D** Calceoli of flagellum on antenna 1 **E** Antenna 2 **F, G** Calceoli of peduncular article 5 and flagellum on antenna 2 **H** Upper lip **I** Lower lip **J** Maxilla 1 **K** Setae of outer plate on maxilla 1 **L** Maxilla 2. Scale bars = 0.1 mm (**C, D, F, G, K**), 0.2 mm (**H–J, L**), 0.5 mm (**A, B, E**).

**Figure 2.**
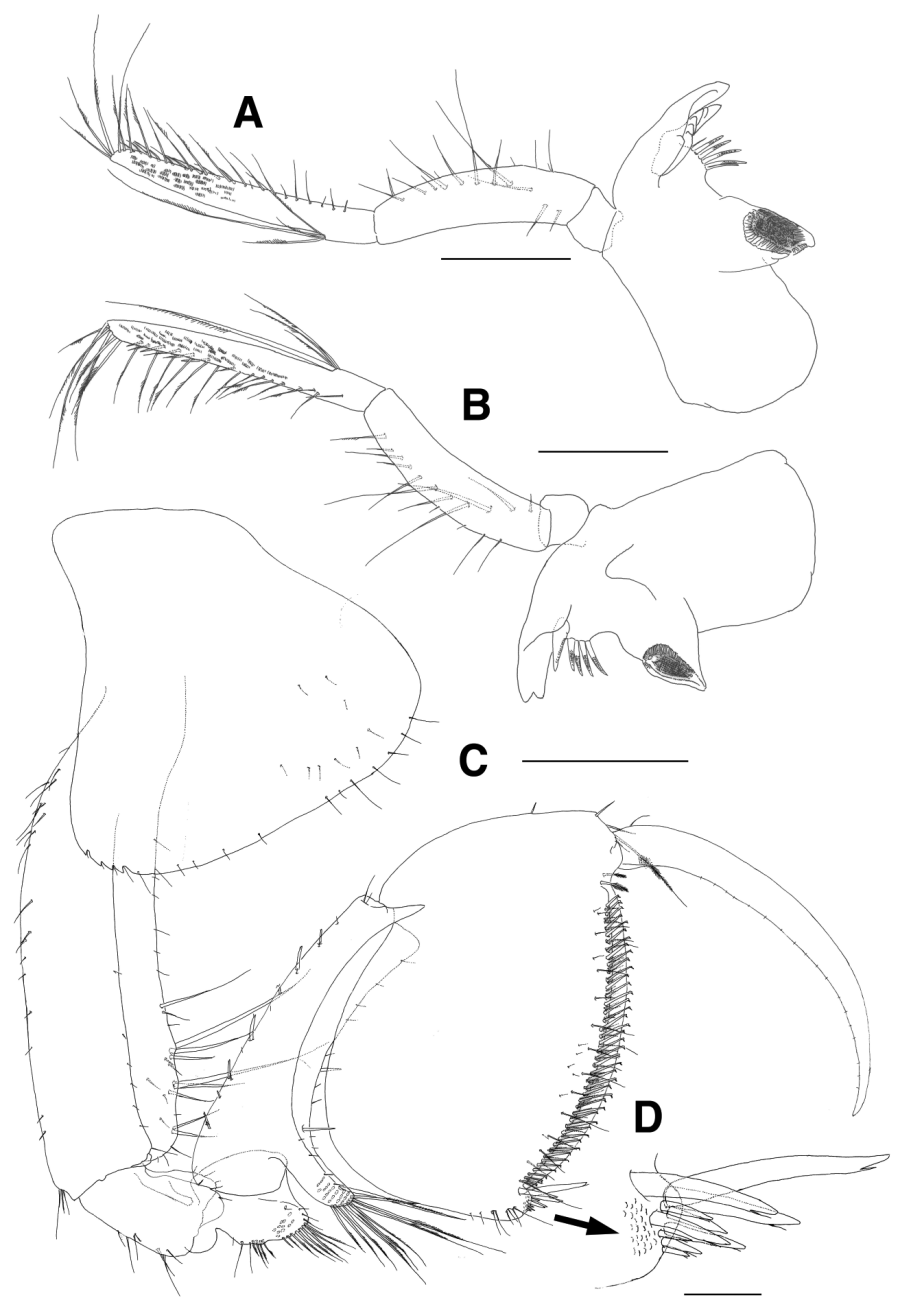
*Eusirus bulbodigitus* sp. n., holotype, male, NIBRIV0000332003, 11.3 mm. Jeju Island, South Korea. **A** Left mandible **B** Right mandible **C** Gnathopod 1 **D** Setae of posterior margin of propodus on gnathopod 1. Scale bars = 0.05 mm (**D**), 0.4 mm (**C**), 0.5 mm (**A, B**).

**Figure 3.**
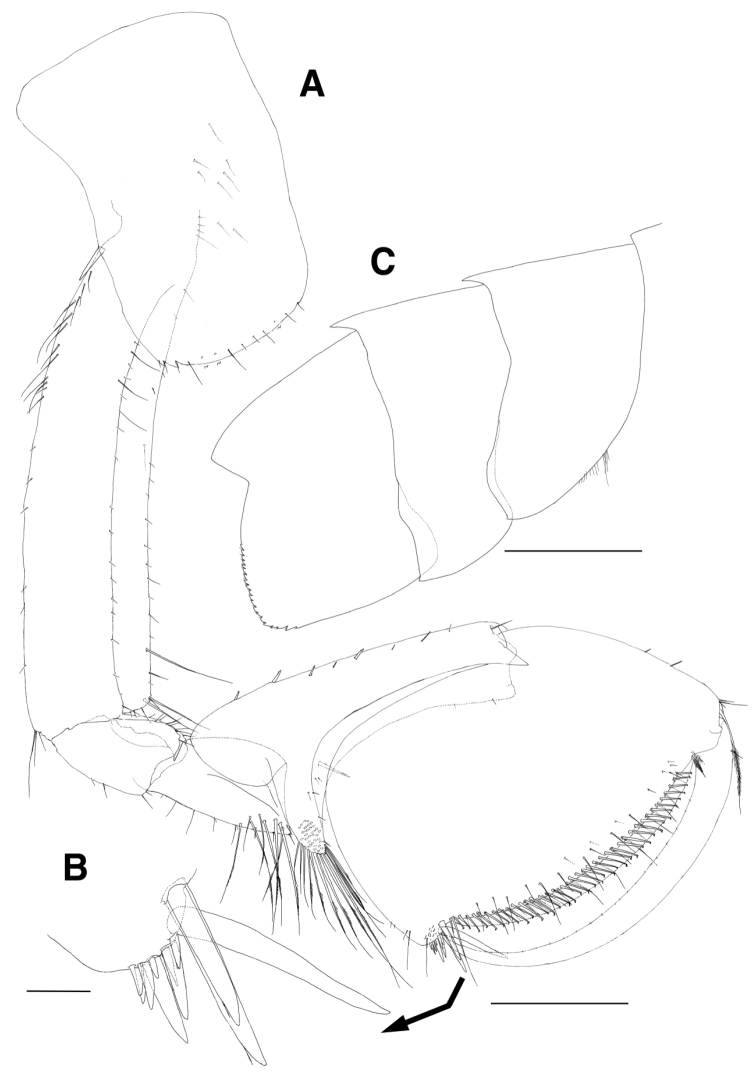
*Eusirus bulbodigitus* sp. n., holotype, male, NIBRIV0000332003, 11.3 mm. Jeju Island, South Korea. **A** Gnathopod 2 **B** Distal setae of posterior margin of propodus on gnathopod 2 **C** Pleonal epimera. Scale bars = 0.05 mm (**B**), 0.4 mm (**A**), 0.5 mm (**C**).

**Figure 4.**
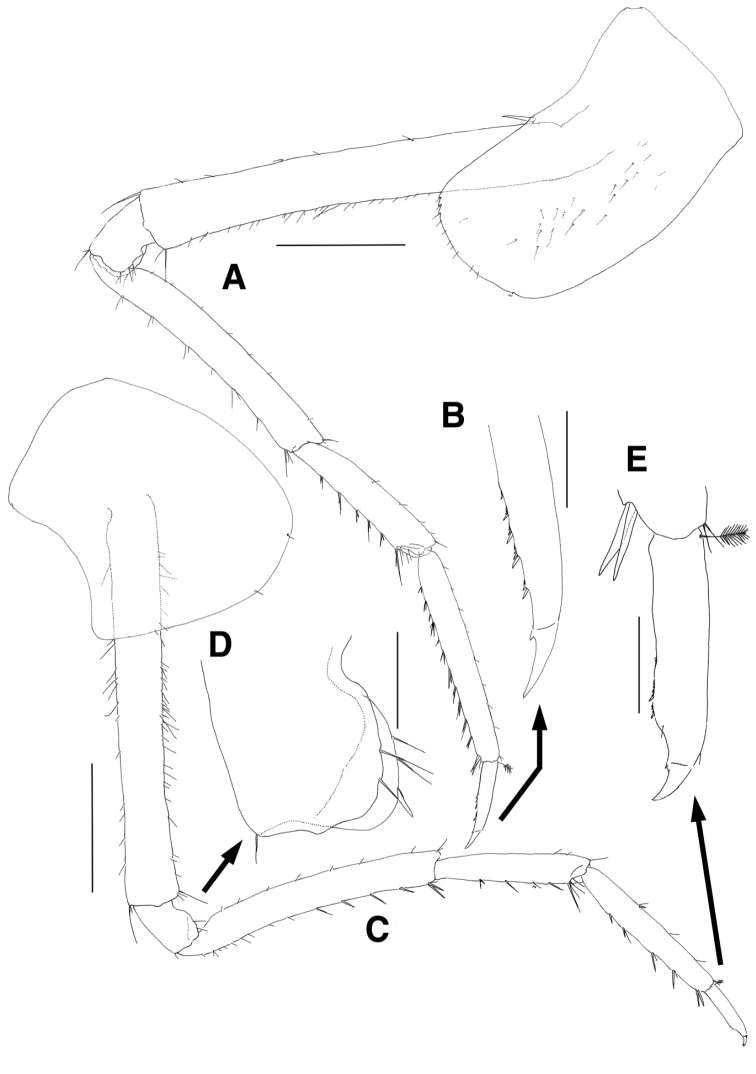
*Eusirus bulbodigitus* sp. n., holotype, male, NIBRIV0000332003, 11.3 mm. Jeju Island, South Korea. **A** Pereopod 3 **B** Dactylus of pereopod 3 **C** Pereopod 4 **D** Ischium of pereopod 4 **E** Dactylus of pereopod 4. Scale bars=0.1 mm (**B, D, E**), 0.5 mm (**A, C**).

**Figure 5.**
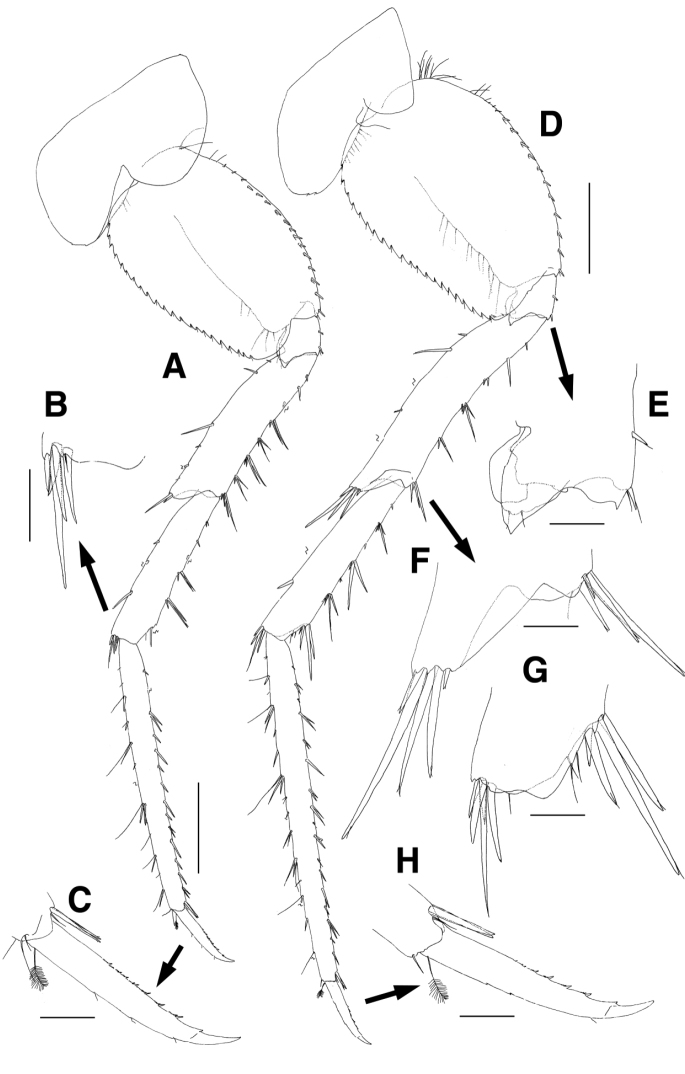
*Eusirus bulbodigitus* sp. n., holotype, male, NIBRIV0000332003, 11.3 mm. Jeju Island, South Korea. **A** Pereopod 5 **B** Posterodistal setae of carpus on pereopod 5 **C** Dactylus of pereopod 5 **D** Pereopod 6 **E** Ischium of pereopod 6 **F** Distal part of merus on pereopod 6 **G** Distal part of carpus on pereopod 6 **H** Dactylus of pereopod 6. Scale bars = 0.1 mm (**B, C, E–H**), 0.5 mm (**A, D**).

**Figure 6.**
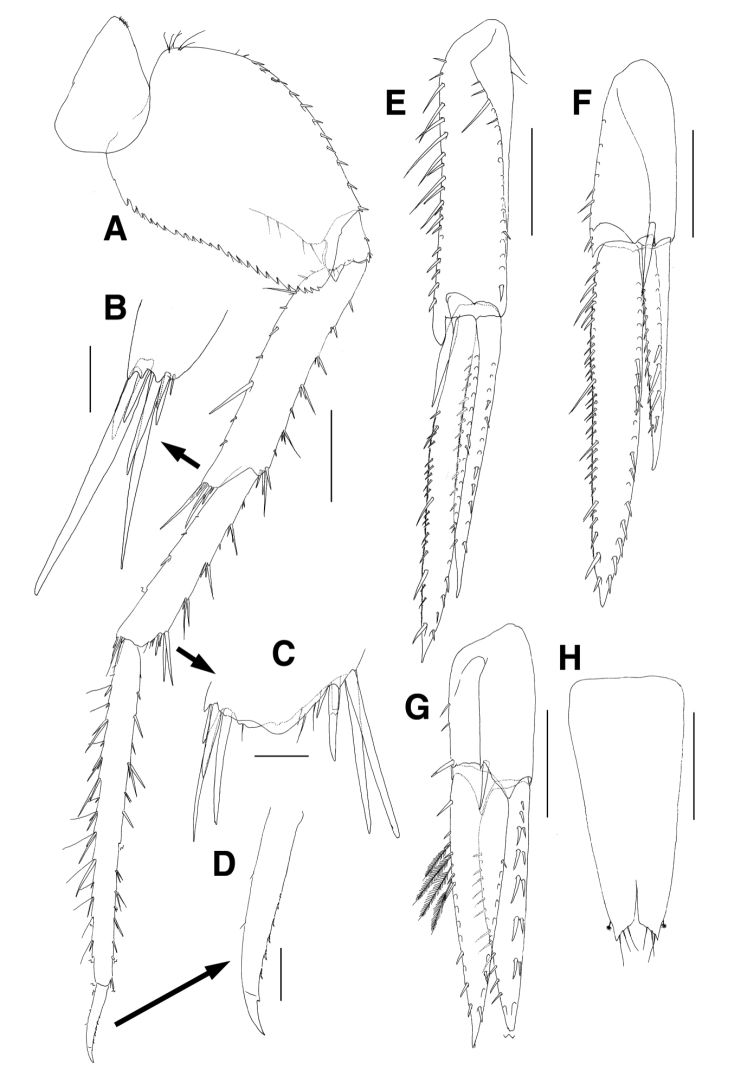
*Eusirus bulbodigitus* sp. n., holotype, male, NIBRIV0000332003, 11.3 mm. Jeju Island, South Korea. **A** Pereopod 7 **B** Posterodistal setae of merus on pereopod 7 **C** Distal part of carpus on pereopod 7 **D** Dactylus of pereopod 7 **E** Uropod 1 **F** Uropod 2 **G** Uropod 3 **H** Telson. Scale bars = 0.1 mm (**B–D**), 0.4 mm (**E–H**), 0.5 mm (**A**).

Figs 1 , 2 , 3 , 4 , 5 , 6

##### Type locality.

Jeju Island, South Korea, 33°29'12"N, 126°57'17"E, sublittoral (average depth 33 m).

##### Material examined.

Holotype: NIBRIV0000332003, adult male, 11.3 mm, collected from the type locality on 30 Nov 2012 by Prof. H-.Y. Soh.

##### Etymology.

The composite epithet of the specific name, *bulbodigitus*, is a combination of Latin *bulbosus* and *digitus*. This name means ‘swollen dactylus’, referring to the shape of the dactylus on pereopod 4.

##### Diagnosis.

Head, lateral cephalic lobe slightly oblique apically; eyes poorly developed. Antenna 1 stout, with peduncular articles 1–3 in length ratio of 4.4:3.8:1.0; peduncular articles 2–3 with 1 robust seta at posterodistal corner subdistally; accessory flagellum uni-articulate. Antenna 2, peduncular article 4 slightly longer than article 5, with calceoli on posterior margin. Maxilla 1, outer plate with 11 dentate setae. Maxilla 2, inner plate broader and larger than outer plate. Left mandible with bi-dentate incisor and 6-dentate lacinia mobilis; right mandible with bi-dentate incisor, lacinia mobilis not split and with 1 row of small dentations apically; palp article 3 with 1 group of 4 serrate setae on lateral margin proximally. Gnathopods stout, “eusiroidean” in form; capus with narrow posterior lobe covered with long serrate setae mediodistally, posterior margin lateral border with acute protrusion at distal corner; propodus wider than long, broad and very deep, with 1 group of defining setae. Pereopod 3 slender, length ratio of merus:carpus:propodus 1.0:0.7:0.9. Pereopod 4, length ratio of merus: carpus:propodus 1.0:0.6:0.7; dactylus with slightly swollen posterior margin. Pereopods 5–7 slender, basis with strong serrations posteriorly. Pleonites 1–2 with acute protrusion dorsodistally; epimeron 3 with 20 serrations posterodistally. Uropod 1, peduncle with 1 enlarged seta at mediodistal corner subdistally; rami lanceolate; outer ramus 0.9 times as long as inner ramus. Uropod 2 0.9 times as long as uropod 1; outer ramus 0.6 times as long as inner ramus. Uropod 3 shortest. Telson shallowly cleft (approximately 1/6 length).

##### Description of holotype male.

Head (Fig. 1A), rostrum distinct, moderate; lateral cephalic lobe concave and slightly oblique apically; antennal sinus not deep; eyes reniform, poorly developed, with boundary composed of separated ommatids.

Antenna 1 (Fig. 1B–D) stout, with length ratio of 4.4:3.8:1.0 in peduncular articles 1–3; peduncular article 1 stout, anterior margin with 1 row of plumose setae proximally, distal margin serrate medially; peduncular article 2 moderate, with groove at anterodistal corner, with 1 robust seta at posterodistal corner subdistally, distal margin serrate; peduncular article 3 short, with 1 robust seta at posterodistal corner; accessory flagellum uni-articulate, as long as 1^st^ proximal article of flagellum, with 2 simple setae subapically and 1 plumose seta apically; flagellum 50-articulate, 1.5 times as long as peduncular articles 1–3 combined, proximal article longest, posterodistal aesthetascs or calceoli present irregularly.

Antenna 2 (Fig. 1E–G) shorter than antenna 1; peduncular articles 4–5 stout, armed with simple, robust and plumose setae of various combinations; peduncular article 4 with 2 calceoli on posterior margin distally, possessing groove at anterodistal corner; peduncular article 5 slightly shorter than peduncular article 4, posterior margin with groups of calceoli and setae; flagellum slightly shorter than peduncular articles 4–5 combined, 32-articulate, with calceoli posteodistally from 1^st^ to 14^th^ articles.

Upper lip (Fig. 1H) globular, apex convex and weakly produced, covered with marginal and submarginal fine setae.

Lower lip (Fig. 1I), inner lobe weak, covered with marginal and submarginal fine setae; outer lobe subovoid, round distally, covered with fine setae on apex and medial margin; mandibular process short.

Maxilla 1 (Fig. 1J, K), outer plate with 11 dentate setae on apical margin; palp long, slender, beyond apical setae of outer plate, palp article 1 elongate, 0.8 times as long as outer plate, palp article 2 with 1 row of 12 setae along apex and mediodistal margin.

Maxilla 2 (Fig. 1L), inner plate ovoid, broader, larger than outer plate, lined with 17 marginal setae from apex to distal half of medial margin, with 4 plumose setae subdistally and 6 facial setae on medial margin; outer plate with 18 setae on apical margin.

Left mandible (Fig. 2A) with bi-dentate incisor and 6-dentate lacinia mobilis; accessory setal row composed of 2 simple and 4 dentate setae; palp 3-articulate, palp article 1 shortest, palp article 2 slightly curved, with 1 row of 11 setae on surface obliquely, palp article 3 1.2 times as long as palp article 2, gradually slender distally, covered with several rows of minute setae on distal surface, with 1 group of 4 serrate setae on lateral margin proximally and longest seta among them reaching apex of palp article 3, lined with simple and serrate setae on medial margin, apex oblique and with 5 long serrate setae. Right mandible (Fig. 2B) with bi-dentate incisor, lacinia mobilis not split and with 1 row of small dentations apically; accessory setal row composed of 3 dentate setae; molar triturative, columnar; palp similar to that of left mandible.

Gnathopod 1 (Fig. 2C, D) stout, strongly subchelate, “eusiroidean” in form; coxa subtriangular, slightly expanded anteroventrally, with 13 submarginal setae on ventral margin irregularly, with 4 small notches bearing 1 minute seta and more expanded backwards posterodistally, with 9 setae on medial surface; basis steady in width, anterior margin lateral border shallowly lobate distally bearing 1 minute seta, with 6 short setae on distal half, medial border weakly lobate unevenly bearing 4 groups of elongate setae on distal 2/3, posterior margin lined with 19 submarginal 4 setae, with 1 group of 3 setae distally; ischium largely lobate, anterior margin lateral border expanded distally, with 3 minute setae, medial border lobate, with 3 minute setae distally, posterior margin with 7 setae; merus subrectangular, as long as ischium, forming groove and broadly lobate on anterior margin, posterior margin lined with serrate setae, with 12 serrate setae on medial surface posterodistally; carpus elongate and slender, 0.8 times as long as basis in length of anterior margin, anterior margin lined with regularly spaced robust and simple setae, carpal lobe narrow, covered with elongate serrate setae mediodistally, posterior margin lateral border with acute protrusion distally, medial border broadly lobate; propodus wider than long, broad, very deep, shorter than that of carpus in length of anterior margin, with 1 plumose seta and 2 simple setae at anterodistal corner, proximal 2/3 of posterior margin fitting well to distal groove of carpus and with setae distally, with 1 group of 10 defining setae, palm long, convex, lined with numerous crooked setae submarginally, with 1 pair of plumose setae distally; dactylus falcate, stout, long, fitting palm, lined with minute setae along inner margin.

Gnathopod 2 (Fig. 3A, B) similar to gnathopod 1, stout, strongly subchelate, also “eusiroidean” in form; coxa rectangular, with 2 small notches bearing 1 minute seta at posterodistal corner, with 1 robust seta on posterior margin, with 8 setae on medial surface, ventral margin convex, with 10 submarginal setae; basis steady in width, anterior margin lateral border shallowly lobate distally bearing 1 minute seta and with 18 setae, medial border with 1 long and 12 short setae, with 1 pair of elongate setae at anterodistal corner, posterior margin lined with 12 setae on proximal 1/3, remainder just with 6 minute setae, with 1 group of 3 setae at posterodistal corner; ischium largely lobate, expanded distally, anterior margin with 5 minute setae on lateral and medial borders, respectively, posterior margin just with 2 setae, with 1 pair of setae at posterodistal corner; merus subrectangular, slightly longer than ischium, forming groove anteriorly, posterior margin lined with short setae, covered with 7 long serrate setae on posterodistal surface, posterodistal corner weakly produced, with 2 short setae; carpus elongate and slender, 0.7 times as long as basis, anterior margin lined with robust and simple setae regularly, carpal lobe narrow, covered with elongate serrate setae medially, posterior margin lateral border with acute protrusion distally, medial border broadly lobate, with 2 minute setae marginally, with 1 robust seta on medial surface; propodus wider than long, broad, very deep, shorter than that of carpus in length of anterior margin, anterior margin with 2 setae, with 1 plumose and 3 simple setae at anterodistal corner, proximal 2/3 of posterior margin fitting well to distal groove of carpus, with 1 group of 11 defining setae, palm long, convex, lined with numerous crooked setae submarginally, with 1 pair of plumose setae proximally; dactylus falcate, stout, long, fitting palm, lined with minute setae along inner margin.

Pereopod 3 (Fig. 4A, B) slender, length ratio of merus:carpus:propdus 1.0:0.7:0.9; coxa rectangular, convex, with 8 submarginal setae on ventral margin, expanded backwards, with 3 small notches bearing 1 minute seta at posteroventral corner, with 1 robust seta on posterior margin, with 23 setae on medial surface; basis linear and elongate, 1.4 times as long as coxa, anterior margin a little lobate distally, lined with 18 single and 2 pairs of setae on distal 2/3, with 1 pair of setae at anterodistal corner, posterior margin with 5 setae, with 1 long seta at posterodistal corner; ischium lobate distally, with 3 minute setae on lateral and medial borders of anterior margin, respectively, with 1 pair of unequal setae at posterodistal corner; merus 0.6 times as long as basis, anterior margin with 7 minute setae, anterodistal corner weakly produced, with 1 pair of unequal setae, posterior margin with 6 pairs of short setae, with 1 group of 3 setae at posterodistal corner; carpus slightly dilated distally, 0.5 times as long as merus, anterior margin with 4 minute setae marginally and 1 seta distally, posterior margin with 5 pairs of unequal setae marginally and 1 pair of setae distally, distal margin obliquely truncated posteriorly, with 2 setae on lateral and medial borders, respectively; propodus, 0.9 times as long as merus, anterior margin with 8 minute setae, with 1 pair of minute setae at anterodistal corner, posterior margin with 11 groups of setae, with 1 pair of locking setae and 1 seta at posterodistal corner; dactylus elongate, 0.3 times as long as propodus, curved distally, with 5 rows of small teeth and 1 subdistal protrusion on distal half of posterior margin.

Pereopod 4 (Fig. 4C–E), length ratio of merus:carpus:propdus 1.0:0.6:0.7; coxa shorter than that of pereopod 3, broadly produced backwards and absent of robust seta on posterior margin; dactylus, posterior margin slightly swollen, parallel to anterior margin until distal 3/4 possessing 3 rows of small teeth, drastically diminished and forming falcation distally; shapes of other articles similar to those of pereopod 3.

Pereopod 5 (Fig. 5A–C), coxa bilobate subequally, anterior lobe slightly larger than posterior lobe, both expanded posteroventrally, posterior lobe with 2 subacute teeth posteroventrally; basis ovoid, convex anteriorly, anterior margin with 4 long setae proximally and lined with 15 setae, with 1 small acute protrusion and paired robust and minute setae at anterodistal corner, posterior margin lateral border moderately expanded and with 22 strong serrations bearing 1 minute seta, medial border with 2 setae distally and with 3 setae at angulate distal corner; ischium short, anterior margin with 1 seta, with 1 small acute protrusion and paired robust and minute setae at anterodistal corner, posterior margin lateral border largely lobate, its apex acute and slightly lurched distally, medial border also lobate but not produced distally; merus 0.7 times as long as basis, anterior margin lined with 3 single setae and 4 groups of setae, with 1 group of 4 setae at anterodistal corner, posterior margin slightly expanded, with 5 setae irregularly, with 1 pair of unequal setae at produced posterodistal corner; carpus as long as merus, anterior margin with 5 groups of setae, anterodistal corner obliquely truncated, with 1 group of 8 setae, posterior margin with several setae and 1 group of 6 setae distally; propodus linear and elongate, 1.8 times as long as carpus, anterior margin with 11 single and 3 paired setae, with 1 pair of locking setae and 1 lateral seta distally, posterior margin setose irregularly, with 3 setae at posterodistal corner; dactylus also elongate, 0.3 times as long as propodus, curved distally, with 7 rows of small teeth and 1 subdistal protrusion on distal 2/3 of anterior margin, with 3 minute setae on posterior margin.

Pereopod 6 (Fig. 5D–H) longer than pereopod 5; coxa bilobate, anterior lobe smaller, posterior lobe more dilated posteroventrally, with 2 small notches bearing 1 minute seta; basis ovoid, anterior margin with 15 setae proximally and lined with 12 setae, with 1 small acute protrusion, 1 robust and 1 simple seta at anterodistal corner, posterior margin lateral border moderately expanded, with 21 strong serrations bearing 1 minute seta, medial border with 10 setae on distal half; ischium short, anterior margin with 1 seta, with 1 seta and 1 small acute protrusion at anterodistal corner, posterior margin lateral border largely lobate, its apex acute and slightly lurched distally, medial border also lobate but not produced distally; merus as long as basis, anterior margin slightly concave, armed with setae of various combination, posterior margin broadly expanded, with 5 setae, posterodistal corner produced, with 1 group of 5 unequal setae; carpus 0.9 times as long as merus, anterior margin with 2 single, 1 paired short setae and 3 groups of setae, anterodistal corner obliquely truncated, with 1 group of 4 robust setae and 1 medial, 2 lateral setae, posterior margin with 2 setae, with 1 group of 4 setae at posterodistal corner; propodus linear and elongate, 1.8 times as long as carpus, anterior margin with 10 single and 7 paired setae, with 1 pair of locking setae and 1 lateral seta at anterodistal corner, posterior margin densely setose irregularly, with 1 seta at posterodistal corner; dactylus also elongate, 0.2 times as long as propodus, curved distally, with 6 rows of small teeth and 1 subdistal protrusion on distal 2/3 of anterior margin, with 2 minute notches on posterior margin.

Pereopod 7 (Fig. 6A–D) longer than pereopod 6; coxa unilobate, convex ventrally, slightly dilated posteroventrally, with 1 group of minute setae on anterior margin proximally; basis subovoid, anterior margin with 6 setae proximally, lined with 2 minute and 11 robust setae, with 1 small acute protrusion and 2 setae at anterodistal corner, posterior margin lateral border more expanded proximally, with 23 strong serrations bearing 1 minute seta, medial border with 4 setae; ischium short, anterior margin with 1 seta, with 1 seta and 1 small acute protrusion at anterodistal corner, posterior margin lateral border largely expanded, slightly lurched distally bearing acute apex, medial border also lobate but not produced distally, with 3 setae at distal corner; merus as long as basis, anterior margin weakly setose, with 1 group of 4 setae at anterodistal corner, posterior margin broadly expanded, with 7 short and 2 elongate setae, posterodistal corner produced distally, with 1 group of 8 unequal setae; carpus 0.8 times as long as merus, slightly dilated distally, anterior margin setose, anterodistal corner obliquely truncated, with 1 group of 5 setae, 2 lateral and 1 medial setae, posterior margin setose, with 1 group of 5 setae at posterodistal corner; propodus linear, elongate, 1.6 times as long as carpus, anterior margin setose, with 1 pair of locking setae and 1 lateral seta at anterodistal corner, posterior margin densely setose irregularly; dactylus also elongate, 0.2 times as long as propodus, curved distally, with 6 rows of small teeth and 1 subdistal protrusion on distal 2/3 of anterior margin, with 2 minute setae on posterior margin.

Pleon (Fig. 3C), lateral ledge absent; pleonite 1 with acute protrusion dorsodistally, epimeron 1 dilated posteroventrally with subacute corner, with 1 group of 13 setae anteriorly on ventral margin; pleonite 2 also with acute protrusion dorsodistally, epimeron 2 not larger than epimeron 1, convex ventrally, weakly produced posteroventrally; pleonite 3 without acute protrusion on dorsal margin, epimeron 3 largest, flattened ventrally, posterior margin expanded backwards and with 20 serrations bearing 1 minute seta on distal half.

Uropod 1 (Fig. 6E) slender, rami lanceolate; peduncle with 18 lateral and 20 medial setae dorsally, with 1 blunt protrusion and 1 enlarged seta at mediodistal corner subdistally; inner ramus as long as peduncle, medial margin weakly serrate, with 16 short and 4 elongate setae dorsally, lateral margin with more than 19 setae dorsally; outer ramus 0.9 times as long as inner ramus, medial margin weakly serrate, with 15 dorsally, lateral margin with 13 setae dorsally.

Uropod 2 (Fig. 6F) 0.9 times as long as uropod 1, rami lanceolate; peduncle 0.7 times as long as that of uropod 1, with 9 medial setae dorsally, weakly produced mediodistally, with 1 elongate seta at laterodistal corner; inner ramus 1.6 times as long as peduncle, lateral margin with more than 21 setae, medial margin serrate, with 34 setae dorsally; outer ramus 0.6 times as long as inner ramus, with 8 lateral setae dorsally, medial margin weakly serrate, with 12 setae dorsally.

Uropod 3 (Fig. 6G) shortest; peduncle 0.8 times as long as that of uropod 2, with acute protrusion on both laterodistal and mediodistal corners, with 2 medial setae dorsally, with 1 robust seta at both mediodistal and laterodistal corners; rami lanceolate; inner ramus 2.0 times as long as peduncle, with robust and plumose setae medially; outer ramus slightly shorter than inner ramus, with 2 single and 5 paired setae dorsally along lateral margin, medial margin with 15 setae.

Telson (Fig. 6H) shallowly cleft (approximately 1/6 length), each apex acute, with 3 setae medially on obliquely truncated margin, with 1 subdistal plumose seta laterally.

##### Remarks.

*Eusirus bulbodigitus* sp. n. shares the characteristic of a mandibular palp bearing a group of setae laterally on the 3^rd^ article with five known species: *Eusirus abyssi* Stephensen, 1944; *Eusirus columbianus* Bousfield & Hendrycks, 1995; *Eusirus hirayamae* Bousfield & Hendrycks, 1995; *Eusirus laticarpus* Chevreux, 1906; and *Eusirus parvus* Pirlot, 1934 (Chevreux 1906, Pirlot 1934, Stephensen 1944, Bousfield and Hendrycks 1995). However, *Eusirus abyssi* is readily discriminated from *Eusirus bulbodigitus* sp. n. by the absence of eyes, the massive carpal lobes of gnathopods 1 and 2, the elongate articles of pereopod 3, and the presence of dorsal teeth on urosomite 1 (Stephensen 1944). Both *Eusirus columbianus* and *Eusirus laticarpus* can be differentiated from *Eusirus bulbodigitus* sp. n. by their developed eyes and the protruding apical margin of each lobe on telson (Chevreux 1906, Bousfield and Hendrycks 1995). In addition *Eusirus columbianus* represents additional differences as follows: (1) the accessory flagellum of *Eusirus columbianus* is longer than that of *Eusirus bulbodigitus* sp. n.; (2) the inner plate of maxilla 2 in *Eusirus columbianus* is smaller than that of *Eusirus bulbodigitus* sp. n.; (3) among the lateral setae on mandibular palp article 3, the longest seta is not reaching at distal end of article 3 in *Eusirus columbianus* (vs. reaching in *Eusirus bulbodigitus* sp. n.); (4) the merus, carpus and propodus of pereopod 4 are slightly longer than those of pereopod 3 in *Eusirus columbianus* (vs. slightly shorter in *Eusirus bulbodigitus* sp. n.); (5) the posterior margin of pleonal epimeron 3 is serrate entirely in *Eusirus columbianus* (vs. partially serrate on distal half in *Eusirus bulbodigitus* sp. n.); and (6) the lateral margin of outer ramus on uropod 3 is lined with single setae in *Eusirus columbianus* (vs. with paired setae in *Eusirus bulbodigitus* sp. n.) (Bousfield and Hendrycks 1995). *Eusirus bulbodigitus* sp. n. is very similar to *Eusirus parvus*. However, it can be distinguishable from the latter in following characters combined: (1) the eyes are poorly developed in *Eusirus bulbodigitus* sp. n. (vs. well-developed in *Eusirus parvus*); (2) the mandibular palp of *Eusirus bulbodigitus* sp. n. is slender than that of *Eusirus parvus*; (3) the longest seta of mandibular palp article 3 is reaching to the end of article in *Eusirus bulbodigitus* sp. n. (vs. not reaching in *Eusirus parvus*); (4) the articles of pereopods in *Eusirus parvus* are slightly longer than those of *Eusirus bulbodigitus* sp. n.; and (5) the inter-ramal process of uropod 1 is absent in *Eusirus bulbodigitus* sp. n. (vs. present in *Eusirus parvus*) (Pirlot 1934). *Eusirus bulbodigitus* sp. n. from Korean waters also closely resembles *Eusirus hirayamae* from Japanese waters. However, it can be clearly distinguished from the latter with the combination of the following characteristic features: (1) the lateral border of carpus on ganthopod 2 is acutely produced posterodistally (vs. not produced and rounded in *Eusirus hirayamae*); (2) both lengths of the carpus and propodus on pereopod 4 are slightly reduced (merus:carpus:propodus = 1.0:0.6:0.7) compared to those of pereopod 3 (merus:carpus:propodus = 1.0:0.7:0.9), but they are not reduced in pereopod 4 of *Eusirus hirayamae* (merus:carpus:propodus = 1.0:0.7:1.0 in both pereopods 3 and 4); (3) on pereopod 4, the proximal three-quarters of posterior margin of the dactylus is swollen in *Eusirus bulbodigitus* sp. n. (vs. moderate in *Eusirus hirayamae*); (4) the dactylus of pereopods 3 and 5–7 is more elongate and slender in *Eusirus bulbodigitus* sp. n. (vs. shorter and thicker in *Eusirus hirayamae*); (5) the posterior margin of dactylus on pereopods 3–7 has rows of small teeth in *Eusirus bulbodigitus* sp. n. (vs. no teeth in *Eusirus hirayamae*), (6) the setations of articles on pereopods 3–7 in *Eusirus bulbodigitus* sp. n. are weaker than those in *Eusirus hirayamae*; (7) the lateral surfaces of pleonal epimera 1–3 are not covered with setae anteroventrally in *Eusirus bulbodigitus* sp. n. (vs. densely covered with setae in *Eusirus hirayamae*); and (8), the posterior margin of pleonal epimeron 3 is serrated in the distal half (vs. serrations occur along the whole posterior margin in *Eusirus hirayamae*) (Bousfield and Hendrycks 1995).

### Keys to the North Pacific species of the genus *Eusirus*

**Table:** 

1	Mandibular palp article 3 with 1 group of lateral setae proximally	**2**
–	Mandibular palp article 3 without lateral setae proximally	**4**
2	Eyes developed; pereopod 4, articles moderate (not reduced than those of pereopod 3)	**3**
–	Eyes poorly developed; pereopod 4, articles slightly reduced than those of pereopod 3	***Eusirus bulbodigitus* sp. n.**
3	Coxa 6, posterior lobe not expanded posterodistally; coxa 7 bilobed; uropod 1, peduncle with developed inter-ramal process	***Eusirus columbianus* Bousfield & Hendrycks, 1995**
–	Coxa 6, posterior lobe expanded posterodistally; coxa 7 unilobed; uropod 1, peduncle without developed inter-ramal process	***Eusirus parvus* Pirlot, 1934**
4	Pleonal epimeron 3 strongly serrate along posterior margin; telson cleft deeply	**5**
–	Pleonal epimeron 3 not serrate or weakly serrate along posterior margin; telson not deeply cleft (slightly notched apically)	**6**
5	Coxa 1 moderately expanded backwards; uropod 3, inner ramus slightly shorter than outer ramus; telson cleft apically approx. 1/2 length	***Eusirus cuspidatus* Krøyer, 1845**
–	Coxa 1 strongly expanded backwards; uropod 3, rami subequal in length; telson cleft apically approx. 1/3 length	***Eusirus hirayamae* Bousfield & Hendrycks, 1995**
6	Antenna 2, peduncular articles slender; pereopod 5, basis broadly expanded posteriorly; pereopod 7, basis narrowly expanded posteriorly; telson elongate	***Eusirus bathybius* Schellenberg, 1955**
–	Antenna 2, peduncular articles stout; pereopod 5, basis weakly expanded posteriorly; pereopod 7, basis convex posteroproximally; telson triangular	***Eusirus fragilis* Birstein & Vinogradov, 1960**

## Supplementary Material

XML Treatment for Eusirus bulbodigitus
